# Impact of Maternal Food Restriction on Heart Proteome in Appropriately Grown and Growth-Restricted Wistar—Rat Offspring

**DOI:** 10.3390/nu13020466

**Published:** 2021-01-30

**Authors:** Andreas Zouridis, Antigoni Manousopoulou, Anastasios Potiris, Polyxeni-Maria Sarli, Leon Aravantinos, Panagiota Pervanidou, Efthymios Deligeoroglou, Spiros D. Garbis, Makarios Eleftheriades

**Affiliations:** 1 2nd Department of Obstetrics and Gynecology—Aretaieion Hospital, National and Kapodistrian University of Athens, 11 528 Athens, Greece; apotiris@gmail.com (A.P.); elinasarli@gmail.com (P.-M.S.); leonaras@hotmail.com (L.A.); deligeoroglou@yahoo.gr (E.D.); makarios@hotmail.co.uk (M.E.); 2 City of Hope National Medical Center, Beckman Research Institute, Duarte, CA 91010, USA; amanousopoulou@coh.org; 3 Unit of Developmental and Behavioral Pediatrics—1st Department of Pediatrics, National and Kapodistrian University of Athens, 11 527 Athens, Greece; nenyperva@gmail.com; 4 Faculty of Medicine, Institute for Life Sciences, University of Southampton, Southampton SO17 1BJ, UK; s.d.garbis@proteas-bioanalytics.com

**Keywords:** FGR, IUGR, heart, cardiac, cardiovascular, food restriction, fetal programming

## Abstract

Objective: Fetal growth restriction is associated with increased postnatal cardiovascular morbidity. The alterations in heart physiology and structure caused by in utero nutrient deprivation have not been extensively studied. We aim to investigate the impact of maternal food restriction on the cardiac proteome of newborn rats with normal (non-fetal growth-restricted (FGR)) and reduced (FGR) birth weight. Methods: On day 14 of gestation, 10 timed pregnant rats were randomized into two nutritional groups: (a) Standard laboratory diet and (b) 50% global food restriction. Pups born to food-restricted mothers were subdivided, based on birthweight, into fetal growth-restricted (FGR) and non-FGR, while pups born from normally nourished mothers were considered controls. Rat neonates were euthanized immediately after birth and the hearts of 11 randomly selected male offspring (*n* = 4 FGR, *n* = 4 non-FGR, *n* = 3 control group) were analyzed using quantitative proteomics. Results: In total, 7422 proteins were quantified (q < 0.05). Of these, 1175 were differentially expressed in FGR and 231 in non-FGR offspring vs. control with 151 common differentially expressed proteins (DEPs) between the two groups. Bioinformatics analysis of DEPs in FGR vs. control showed decreased integrin and apelin cardiac fibroblast signaling, decreased muscle contraction and glycolysis, and over-representation of a protein network related to embryonic development, and cell death and survival. Conclusion: Our study illustrates the distinct proteomic profile of FGR and non-FGR offspring of food-restricted dams underlying the importance of both prenatal adversities and birth weight in cardiac physiology and development.

## 1. Introduction

Barker’s epidemiological studies in the late-80s linked poor nutrition in early life with an increased mortality rate of ischemic heart disease [1], and showed that birth weight was inversely correlated to systolic blood pressure later in life [2]. In 1991, Hales and Barker first introduced the “Thrifty phenotype hypothesis” to explain the link between the inadequate early nutrition and the development of type 2 diabetes mellitus later in life. According to this hypothesis, the thrifty phenotype could be the outcome of “fetal programming” under unfavorable conditions, causing structural and functional alterations that help the fetus to cope with in utero adversities, predisposing however to adult morbidity [3,4].

Maternal nutrition is crucial for placental-fetal development [5]. Both experimental [6] and clinical studies [7,8] show that maternal caloric restriction results in small for gestational-age fetuses (SGA) and low-birth-weight infants (LBW). According to the current classification [9], SGA fetuses and LBW infants of undernourished mothers are considered growth-restricted (fetal growth-restricted (FGR)), as intrauterine nutrient deficiency impedes them from reaching their growth potential. Although FGR and LBW are distinct conditions, many researchers use them as synonyms to describe birthweight below the 10th or 5th percentile [9]. According to a consensus in definitions of fetal growth restriction, fetal size below the 3rd percentile (approximating 2 SD) can be used as an isolated criterion to define FGR at any gestational age [10]. The above definition has been adopted by the latest ISUOG guidelines for FGR diagnosis and management [11].

Low birthweight predisposes to ischemic heart disease [12,13,14], stroke [12,14], and atrial fibrillation [15,16]. Males’ offspring cardiovascular systems seem to be more vulnerable to an intrauterine insult compared to females [17,18]. Association between low birth weight and cardiovascular disease was initially considered secondary to obesity, diabetes mellitus, and hypertension [19]. However, the presence of cardiovascular rearrangements at growth-restricted individuals during infancy and childhood [20] suggests a direct effect of growth restriction to the cardiovascular system.

The aim of our study was to compare the global heart proteomic profile between three groups of newborn male rats: Offspring of normally nourished mothers, growth-restricted offspring of food-restricted mothers, and appropriately grown offspring of food-restricted mothers. We intend to investigate whether maternal food restriction during late gestation affects the offspring cardiac proteome irrespective of birth weight, and propose possible pathophysiological mechanisms of cardiac fetal programming.

## 2. Materials and Methods

### 2.1. Animal Model

Ten (*n* = 10) nulliparous timed pregnant Wistar Rats (RjHan:WI, Janvier Labs, France) [21] were housed individually in 36 × 20 × 14 cm breeding boxes. The housing conditions were standard (22–23 °C temperature, 55–65% humidity, 12 h dark-light cycle starting at 9:00) and the rats were fed a formula containing 18.5% protein (4RF21, Mucedola S.r.l., Italy). They had *ad libitum* access to food and water until the 14th day of gestation. On the 14th day of gestation, pregnant rats were randomly assigned to either a control (*n* = 4) or starved (*n* = 6) group. Rats of the control group had *ad libitum* access to food, while in the starved group, food was restricted by 50% until delivery [6]. Both groups had free access to fresh water.

Moderate food restriction between the 15th and 21st day of gestation significantly decreases birthweight in contrast with caloric manipulation in early gestation [6]. We were measuring the control group’s food intake on a daily basis. From day 15th onwards, rats belonging to the starved group were given half the amount of food that was on average consumed by the control group rats, based on measurements taken place the day before.

All rats delivered spontaneously and offspring were immediately weighted and separated from their mothers to avoid cannibalism [22] and breastfeeding. Within an hour from birth, pups were anesthetized using inhaled sevoflurane [23], and euthanized. After euthanasia, the fetal heart was removed, cleaned from blood with phosphate-buffered saline, and stored at −80 °C.

Pups born to mothers that received a standard laboratory diet throughout pregnancy were considered as controls. Offspring of the starved group with a birthweight more than two standard deviations (SD) below the mean weight of control neonates were considered as FGR (birth weight < mean birth weight of control group’s offspring −2 × SD), while the remaining pups of starved mothers were characterized as non-FGR (birth weight ≥ mean birth weight of control group’s offspring −2 × SD) [24,25,26].

Litter size, gestation duration, and growth characteristics of mothers, pups, and their organs were compared using the independent-samples *t*-test (IBM SPSS Statistics 22.0). Statistical significance was considered at *p* < 0.05.

All animal procedures were approved by the Directorate of Veterinary Services (protocol number 1211/19-03-2018) and the Ethical Committee of Aretaieion Hospital (protocol number 011/21-11-2017) and were in accordance with both EU and National legislation.

### 2.2. Proteomic Analysis

#### 2.2.1. Quantitative Proteomics Sample Processing

Hearts from 11 male offspring were used for the quantitative proteomic analysis. The number of samples included in the multiplex proteomics study was dictated by the maximum channels available in the isobaric tag labelling kit [Thermo, Tandem Mass Tag (TMT) 11-plex reagents]. As we have shown before, three biological replicates per experimental group give sufficient statistical power to the multiplex proteomics study due to the genomic homogeneity of the animal model used [27]. The samples were randomly selected from each offspring group: Three from the control, four from the FGR, and four from the non-FGR group. We included three pups from the control group and four of each experimental group as we expected higher phenotypic heterogeneity in the latter.

Heart tissue frozen in liquid nitrogen was homogenized by manual grinding using a mortar and pestle. The powdered tissue was dissolved in lysis buffer (0.5 M triethylammonium bicarbonate, 0.05% sodium dodecyl sulphate) and subjected to pulsed probe sonication (Misonix, Farmingdale, NY, USA). Protein lysates were then centrifuged (4 °C, 15,000 rpm, 10 min) and supernatants were carefully transferred to fresh microcentrifuge tubes. For each sample, the protein content was measured using the BCA protein assay kit (Thermo Scientific, Waltham, MA, USA). The same amount of protein (100 μg) per sample, adjusted to the highest volume using lysis buffer, was reduced, alkylated, and enzymatically digested overnight using trypsin. The resulting peptides were labelled using the TMT reagent kit (11-isobaric tags) and subjected to two dimensional liquid chromatography and mass spectrometry (2D LC-MS) analysis as reported previously [28,29,30].

#### 2.2.2. Database Searching

Proteome Discoverer 1.4 was used for target decoy search of the unprocessed raw data files. We searched against the UniProtKB and TrEMBL rattus norvegicus database (release date July 2018) using the following parameters: No more than two missed cleavages, minimum peptide length of six amino acids, precursor mass tolerance 10 ppm, maximum of two dynamic (one equal) modifications of: Deamination (N, Q), oxidation (M), phosphorylation (S, T, Y). TMT 6-plex (K, and peptide N-terminus) and methylthio (C) were set as static modifications. The false discovery rate (FDR) confidence of peptide identification was set at medium (over 95%). The percent co-isolation threshold that excluded peptide quantitation was set at 50. Only abundances from unique peptides were considered for the quantitation of the respective protein. TMT abundances were median-normalized to correct for different protein amounts loaded per channel.

We considered the ratios of FGR and non-FGR groups vs. controls and performed a log2transformation to normalize the distribution [29]. A one-sample *t*-test for FGR and non-FGR vs. control using the Benjamini, Krieger, and Yekutieli (BKY) multiple hypothesis testing correction was applied to identify differentially expressed proteins (DEPs). Significance was set at 0.05. Proteins identified with at least two unique peptides, a BKY corrected one-sample *t*-test *p* < 0.05, and a mean |log2ratio| > 0.6 (1,5-foldchange) were considered differentially expressed. All proteomics data were uploaded at the ProteomeXchange Consortium via the PRIDE partner repository (dataset identifier PXD011407).

#### 2.2.3. Bioinformatics Analysis

To identify direct protein interaction networks and pathways that were enriched in the DEPs, we used Ingenuity Pathway Analysis (IPA) (Qiagen, Hilden, Germany, https://www.qiagenbioinformatics.com/products/ingenuitypathway-analysis) [31]. *p*-Values ≤ 0.05 were considered significant.

## 3. Results

All pregnant rats delivered at term, between the 21st and 22nd day of gestation, with no difference in pregnancy length between the control and the starved group (20.73 ± 0.06 vs. 21.22 ± 0.47 days, *p* = 0.081). Control mothers gave birth to 46 pups (control group) (mean body weight 6.419 g, SD 0.436). According to the aforementioned definitions, among the pups of starved mothers, 34 were FGR (body weight < 5.547 g) and 31 non-FGR (body weight ≥ 5.527 g). There was no statistical significance in post-delivery maternal weight (268.83 ± 26.75 vs. 264.50 ± 10.47 g, *p* = 0.304) and litter size (10.83 ± 1.72 vs. 11.50 ± 1.29 g, *p* = 0.53) between starved and control group. (Table 1).

The neonatal birth weight in the starved group was significantly lower compared to the control group (5.423 ± 0.610 vs. 6.419 ± 0.436 g, *p* < 0.001). Male offspring were heavier than female in the control group (6.659 ± 0.324 vs. 6.2 ± 0.412 g, *p* < 0.001), whereas there was no statistically significant difference in the starved group (5.454 ± 0.744 vs. 5.388 ± 0.410 g, *p* = 0.666).

Maternal food restriction alters offspring’s heart weight at birth, with the food restricted offspring exhibiting lower values compared to controls (0.027 ± 0.006 vs. 0.035 ± 0.008 g, *p* < 0.001), and the effect remains when adjusting for birth weight (0.00507 ± 0.00103 vs. 0.00552 ± 0.00113 g, *p* = 0.043).

For non-FGR offspring, absolute heart weight was higher than FGR (0.030 ± 0.006 vs. 0.025 ± 0.005 *p* = 0.003), with no statistical difference in heart to body weight ratio between groups (0.00498 ± 0.00094 vs. 0.00515 ± 0,00112, *p* = 0.359). On the contrary, brain absolute weight was similar between FGR and non-FGR pups (0.151 ± 0.048 vs. 0.148 ± 0.043, *p* = 0.783), but relative brain weight was significantly higher for FGR compared to non-FGR offspring (0.03157 ± 0.01093 vs. 0.24956 ± 0.00698, *p* = 0.009).

**Figure 1:**
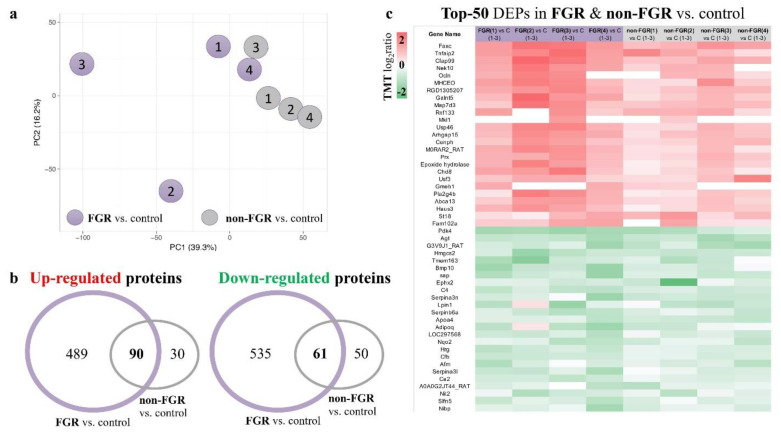
(**a**) Principal component analysis (PCA) of all quantified proteins demonstrated that hearts of fetal growth-restricted (FGR) neonates had a distinct and more heterogeneous proteomic profile compared to non-FGR ones. (**b**) Venn diagram showing overlap of differentially expressed proteins compared to controls in FGR and non-FGR groups. (**c**) Heatmap of top-50 common differentially expressed proteins in both FGR and non-FGR groups vs. control.

The proteomic analysis of male neonatal hearts resulted in the identification of 9412 proteins (peptide level FDR < 0.05), 7422 of which were quantified (Supplementary Table S1). Principal component analysis (PCA) of all quantified proteins demonstrated that hearts of FGR neonates had a distinct and more heterogeneous proteomic profile compared to non-FGR (Figure 1a).

Among the quantified proteins, 5845 were identified with at least two unique peptides. Of these, 579 were up- and 596 proteins were down-regulated in the FGR vs. control group (Supplementary Table S2), whereas 120 were up- and 111 downregulated in the non-FGR compared to control group (Supplementary Table S3). Ninety up- and 61 downregulated proteins were common in both FGR and non-FGR groups vs. control (Figure 1b) (Supplementary Table S4). Figure 1c is a heatmap of the top-50 differentially expressed proteins in the hearts of both FGR and non-FGR neonates vs. control.

**Figure 2:**
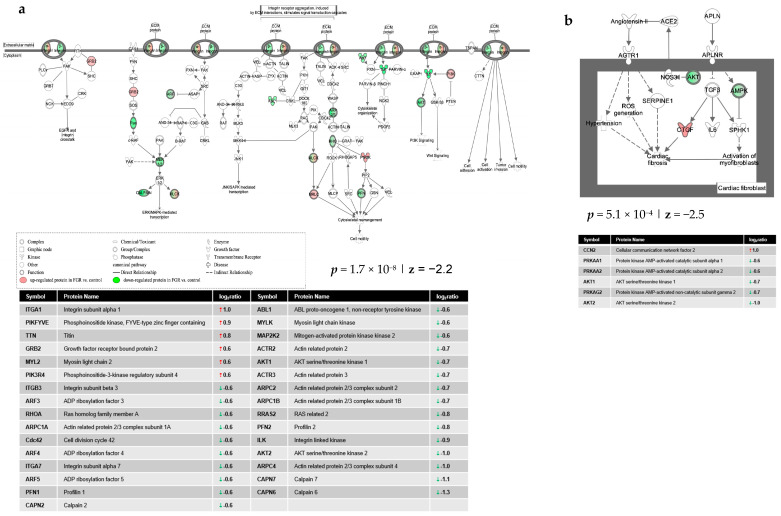
Ingenuity Pathway Analysis of differentially expressed proteins in FGR compared to control showed (**a**) decreased integrin signaling and (**b**) decreased apelin cardiac fibroblast signaling pathway.

**Figure 3:**
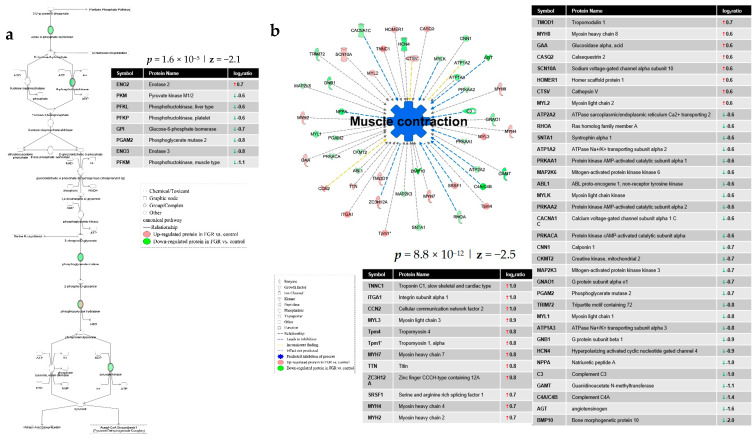
Ingenuity Pathway Analysis of differentially expressed proteins in FGR compared to control showed (**a**) decreased glycolysis and (**b**) decreased muscle contraction.

**Figure 4:**
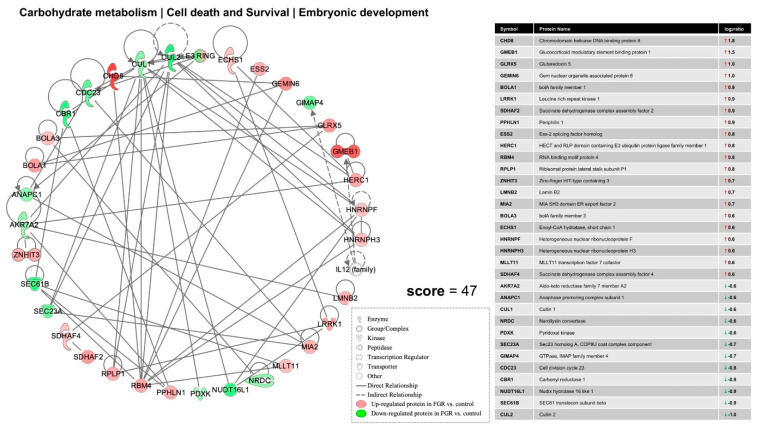
Ingenuity Pathway Analysis of differentially expressed proteins in FGR compared to control showed over-representation of a protein network related to carbohydrate metabolism, cell death and survival, and embryonic development.

Ingenuity Pathway Analysis (IPA) of differentially expressed proteins (DEPs) in FGR compared to control showed inhibition of the integrin signaling (*p* = 1.7 × 10^−8^; z = −2.2) (Figure 2a) and apelin cardiac fibroblast signaling canonical pathways (*p* = 5.1 × 10^−4^; z = −2.5) (Figure 2b). Furthermore, IPA predicted a significant decrease in glycolysis (*p* = 1.6 × 10^−5^; z = −2.1) (Figure 3a) and muscle contraction (*p* = 8.8 × 10^−12^; z = −2.5) (Figure 3b) in the hearts of FGR neonates vs. control. Finally, a protein network related to carbohydrate metabolism, cell death and survival and embryonic development was over-represented in the DEPs of FGR vs. control pups (Score = 47, *n* = 33 proteins) (Figure 4).

## 4. Discussion

The analysis of the weights of offspring tissues showed that both heart and liver weight were reduced in proportion to body weight in FGR compared to non-FGR pups. On the contrary, brain weight did not differ significantly between the two groups (Table 1). That disproportion in growth restriction with the relative preservation of brain growth is characteristic of late, or asymmetrical, FGR where the intrauterine insult happens in late pregnancy [11] (food restriction between the 15th and 21st day of gestation in our model).

Our study is the first one to report the proteomic profiling of hearts in FGR and non-FGR Wistar rat offspring exposed to intrauterine caloric restriction. Maternal undernutrition has been previously shown to differentially affect the metabolic profile [24], body composition [25], and brain proteome [26] of FGR and non-FGR neonates. As indicated by principal compartment analysis, the proteomic profile of FGR and non-FGR hearts is distinguishable, which indicates that maternal food restriction has a different impact in the hearts of neonates with low compared to normal birthweight.

In silico analysis of DEPs in the FGR group revealed reduced integrin and apelin signaling, reduced heart muscle contractility, and reduced glycolysis in fetal hearts compared to the control group, suggestive of unique cardiac adaptations in FGR hearts.

Integrins are transmembrane receptors mediating communication between cardiomyocytes and extracellular matrix, crucial for cardiac development and the response to cardiac disease [32]. The disrupted integrin signaling proposed by IPA in FGR compared to control rats (*p* = 1.7 × 10^−8^; z = −2.2) can be part of the FGR heart pathophysiology. Decreased integrin signaling in FGR neonates can lead to a progressive decrease in postnatal cardiac function [33], partly explaining the origins of persistent subclinical myocardial dysfunction in FGR offspring. In addition, given the crucial role of integrin signaling in myocardial maturation, we can postulate that disruption of that signaling can lead to the immature myocardial phenotype observed in FGR offspring [34].

The apelin/apelin receptor system plays an important role in cardiovascular function, and apelin therapy has beneficial effects in cardiovascular disease [35]. The increased myocardial interstitial fibrosis reported at birth in some animal models of maternal nutrient deprivation [36] can be explained through the reduced apelin fibroblast signaling indicated by our model, as apelin stimulation prevents cardiac fibrosis [37]. In addition, the altered apelin signaling may contribute to the pathophysiology of cardiac remodeling [37] in FGR offspring.

The decrease in myocardial contractility in FGR, but not in non-FGR infants, is consistent with ultrasonographic findings in growth-restricted fetuses and neonates, suggesting subclinical systolic dysfunction [19]. Reduced cardiomyocyte contractility can be attributed to cardiac remodeling, including sarcomere shortening in FGR offspring [20].

Our results suggest that glycolysis is significantly reduced in hearts of FGR infants compared to controls. Experimental studies have shown that placental insufficiency leads to reduced myocardial glucose uptake capacity by the reduction in glucose transporter 1 and 2 expression in FGR offspring [38]. Glucose is the main source of energy in fetal cardiomyocytes, but as the heart matures, metabolism moves toward fatty acids oxidation. However, under stress, heart metabolism moves toward the more energy-efficient glucose again [39].

Glycolysis inhibition may mediate the vulnerability of FGR to heart disease, as it does not allow cardiomyocytes to shift toward glucose consumption in response to stress. This theory is supported by experimental data in rats suggesting that glycolysis inhibition is accompanied by hypertrophy, fibrosis, reduced contractility diastolic dysfunction [40], and predisposition to atrial fibrillation [41].

In our study, maternal food restriction led to reduced heart weight in FGR male offspring. The decrease in FGR heart weight reflects a reduction in total cardiomyocyte number [42], as indicated by previous research. Forty-seven DEPs in our male FGR rat neonates participate in an interesting protein network related to cellular death, survival, and embryonic development. These protein networks could suggest reduced cardiomyocyte number, which make FGR hearts more vulnerable to disease in adulthood, when the cardiomyocyte proliferation potential is limited.

Interestingly, bioinformatics analysis of DEPs in hearts from non-FGR neonates did not reveal any similar alterations in biological functions or protein interaction networks as for the hearts of the FGR pups. It could be assumed that maternal food intake impacts the proteome of neonatal hearts, producing different phenotypes based on birth weight. Maternal food restriction results in unique proteome alterations in FGR hearts, a small subset of common alterations in both FGR and non-FGR hearts, and subtle and less clinically significant alterations in non-FGR hearts.

In conclusion, this study reports the distinct proteomic profile of FGR and non-FGR offspring of food-restricted dams, underlying the importance of both prenatal environment and birth weight in heart physiology and development. Bioinformatics analysis of DEPs in FGR and non-FGR neonates revealed four unique possible pathways of cardiac remodeling in FGR offspring including contractility, apelin and integrin signaling alterations, and glucose metabolism alterations.

## Figures and Tables

**Table 1:** Experimental data.

	Group	Mean	SD	*p*-Value
**Length of gestation (days)**	Starved	21.22	0.47	0.081
	Control	20.73	0.06	
**Litter size (pups)**	Starved	10.83	1.72	0.530
	Control	11.50	1.29	
**Post- delivery maternal weight (g)**	Starved	268.83	26.75	0.304
	Control	264.50	10.47	
**Birth weight (g)**	Starved	5.423	0.610	<0.001
	Control	6.419	0.436	
**Heart weight (g)**	Starved	0.027	0.006	<0.001
	Control	0.035	0.008	
	FGR	0.025	0.005	0.003
	Non-FGR	0.030	0.006	
**Heart to body weight ratio**	Starved	0.00507	0.00103	0.043
	Control	0.00552	0.00113	
	FGR	0.00515	0.00112	0.359
	Non-FGR	0.00498	0.00094	
**Brain weight (g)**	Starved	0.150	0.045	0.001
	Control	0.180	0.044	
	FGR	0.151	0.058	0.783
	Non-FGR	0.148	0.043	
**Brain to body weight ratio**	Starved	0.02826	0.00968	0.905
	Control	0.02806	0.00598	
	FGR	0.03157	0.01093	0.009
	Non-FGR	0.02496	0.00698	
**Liver weight (g)**	Starved	0.245	0.070	0.117
	Control	0.266	0.057	
	FGR	0.211	0.047	<0.001
	Non-FGR	0.280	0.073	
**Liver to body weight ratio**	Starved	0.04498	0.01026	0.112
	Control	0.41753	0.00946	
	FGR	0.04274	0.00743	0.103
	Non-FGR	0.04721	0.01220	

## Data Availability

The data presented in this study are available within the article. All proteomics data are uploaded at the ProteomeXchange Consortium via the PRIDE partner repository (dataset identifier PXD011407).

## Supplementary Materials

The following are available online at https://www.mdpi.com/2072-6643/13/2/466/s1, Table S1: All quantified proteins (peptide level FDR < 0.05), Table S2: DEPs in FGR vs. control (q < 0.05), Table S3: DEPs in non-FGR vs. control (q < 0.05), Table S4: DEPs in hearts of both FGR and non-FGR vs. control (q < 0.05).
